# Identification of five commercial *Eucalyptus* species by SCAR markers development

**DOI:** 10.1186/1753-6561-5-S7-P46

**Published:** 2011-09-13

**Authors:** Karine Kettener, Aletéa Madacki, Tânia Mara Bortoloto, Shinitiro Oda, Celso Luis Marino

**Affiliations:** 1 Genetics Department, Biosciences Institute of Botucatu-SP, Brazil; 2 Suzano Pulp and Paper, Brazil

## 

The genus Eucalyptus native to Australia, had some species introduced in Brazil between the nineteenth and early twentieth century and is currently planted in tropical and subtropical regions of the world. Currently, species of the genus have been highlighted as some of the most important in reforestation projects to provide wood for pulp, paper, energy and solid wood products. Eucalyptus breeders frequently face difficulties to identify closely related species and their hybrids that share common morphological traits, some of them expressed only later in the breeding cycle [1]. Molecular markers could be useful for early identification of species and hybrids. A strategy to identify species specific molecular markers is the use RAPD (Random Amplified Polymorphic DNA) combined with bulked segregant analysis [2]. We used RAPD markers and BSA to find putative species specific markers for five pure species of Eucalyptus: E. tereticornis, E. saligna, E. grandis, E. brassiana and E. urophylla. We tested 112 RAPD primers which generated 187 candidates polymorphic bands, of which 44 have proved reliable after validation carried out by genotyping individually all plants that composed the bulks.A good species-specific candidate RAPD marker was defined as being exclusive to one bulk, have a size between 300 bp and 2000 bp; provide easy visualization in agarose gel and display repeatability in independent assays. The best candidate bands were provided by the Operon primers: AD01, H03, H19, H20, K10, X06, W03, W05 and W07. As expected, although polymorphic between species, none of the RAPD markers were totally exclusive to one species and absent in the others. However by combining sets of two or more RAPD markers with contrasting frequencies among species it was possible to discriminate all species with high confidence. The most informative polymorphic RAPD amplicons were isolated, purified and cloned into pGEM ®-T Easy Vector System I (Promega) and cloned into competent cells of strain DH5α-UltraMAXTM FTTM (Life Tecnlogies, GibcoBRL) for subsequent sequencing and primers design. Based on the sequences obtained a set of SCAR markers was derived after carrying out a temperature gradient test ranging between 52°C and 69°C. The SCAR marker CXT1 allows the identification of E. tereticornis individuals with 80% efficiency. A combination of markers CAS1 and CAS2 or CAS 1 and CWB 1 or CAS2 and CWB 1 allows identifying 90% of the E. saligna individuals. SCAR markers CAG1 combined with CHG1 can identify 100% of the E. grandis individuals; markers CXG1 and CWB1 combined provide 90% identification of E. urophylla and CWB1 is a strong candidate to uniquely identify E. brassiana. These results indicate that the development of SCAR markers from intensive RAPD marker screening can be an inexpensive way to discriminate different species and possibly hybrids.
